# Annotations

**Published:** 1892-01-16

**Authors:** 


### ANNOTATIONS.

**Lady Smokers.**

SEVERAL alarmed enquiries have been addressed to us on the vital question of lady smokers. According to the *Daily Chronicle*, THE HOSPITAL advises ladies to smoke. We, ourselves, who claim to know best, do not quite take that view of the question. In a note in our last week's issue it was stated that the habit of smoking is greatly on the increase among ladies, and that one lady in particular had informed the writer of the note that her medical adviser considered the habit to be actually beneficial to a certain class of women when indulged in moderation. The women whom that particular doctor declared to be benefitted by smoking were women whose nerves were called to bear the strain of a great many small daily worries. But the writer in THE HOSPITAL spoke very cautiously on the subject. "If," said he, "this is the case, we advise all *husbands* who suffer from their wive's overwrought nerves—and these are not a few—to try so simple a remedy." It is thus clear that the persons whom THE HOSPITAL actually advised to smoke were not women, but men, not wives but husbands—and only husbands under peculiar circumstances—that is husbands who have wives with unpleasant nerves. Of course it is open to anybody who so pleases to insist that what is sauce for the gander should also be sauce for the goose; and to that there can be no philosophical objection. We would even go so far as to say that the woman who suffers from her husband's nerves might ask her doctor if smoking were likely to be of service to her. That is to say, as a purely medicinal arrangement there can be no more reason why a woman should not smoke than why she should not medicinally drink brandy. But on the question of taste, THE HOSPITAL shares the opinion of every old-fashioned man and woman in England. As a matter of taste, women ought not to smoke. Indeed, there can be little doubt that the less men smoke the better as a rule. One or two pipes or one cigar a day are quite sufficient for the average town-bred man. Children should never smoke at all; and women should only smoke by intelligent medical advice.

**Influenza Patients in General Hospitals.**

A FEW days ago a gentleman sent out one of his servants, who was suffering from influenza, to seek for admission at one of the London General Hospitals, which we need not name. The young woman was driven up to the hospital door in a cab, but was refused admission on the ground that influenza was an infectious disease. Thus checked at the first hospital the young woman sought a second, a third, and a fourth, with like results, spending no less than six hours in her vain search on one of the coldest days of a cold winter. At last application was made at Guy's, and here humanity was allowed to prevail over science and logic, and the young woman was taken in and kindly cared for as she so much needed to be. We are informed to day by Dr. J. C. Steele, the Medical Superintendent of Guy's Hospital, that the patient's symptoms have rapidly improved under treatment, and that she will be ready to return to her situation in a few days. Much indignation has been expressed in certain quarters at the behaviour of those hospitals that refused admission to the young woman, and more especially did her employer indulge in the language of reprobation. We have no desire to apologise for hospitals when they commit errors or grave faults, but it is eminently for the public interest that the true state of matters should be known and understood. In the first place, then, the case stands thus: That general hospitals are not designed for the treatment of infectious diseases. To introduce infectious cases into the wards of such hospitals would be to run the risk of infecting all the patients of every other class. Thus, the hospital instead of being a house of healing would become a den of pestilence, and patients, other than infectious cases, that were sent there for treatment would be much more likely to be killed than cured. A hospital that admitted all kinds of infectious cases into its general and accident wards would not only be a scientific absurdity but a public danger. So clearly is this recognised by the public authorities, that State-supported and State-managed hospitals for infectious cases are provided not only in London and large provincial towns, but even in smaller towns and outlying country districts. All classes of the people ought to be made aware that the fever hospitals provided by the State are the proper asylums for every infectious disease of every kind that cannot be dealt with by the friends of patients in their own homes. It is impossible that the general hospitals should deal with any such cases without frustrating the very ends for which they exist. We venture to suggest that the gentleman who sent his servant to a general hospital ought to have known that an infectious hospital and not a general hospital was the proper place for her, and should have acted accordingly; and besides, we think that it was not very kind on the part of an employer to send a young woman out in a cab on the mere chance that the first hospital she came to would be able and willing to receive her. A good deal of public ignorance must be allowed for, but it seems to us that if the gentleman in question had exercised reasonable discretion, he would either have gone himself, or sent a trustworthy messenger, to find out a place where hospital accommodation was certain to be obtained before he had asked the young woman to leave her bed. The doors of hospitals stand open night and day, but even hospitals must have rules and regulations; and when those regulations conserve the safety of patients, they should not be set aside on any grounds but those of a vital character.

**Oxford buckles on its Armour.**

IT will give unfeigned pleasure to all those who have a tender care for the sick poor, to know that the managers of the Radcliffe Infirmary at Oxford have now at length fully committed themselves to a scheme for the renovation and modernisation of their hospital. Those who perceive that the practical religion which cares for the helpless is in the very forefront of the noblest things of civilisation, cannot but regard with dismay any divorce between beautiful religious forms and worthy religious deeds. Oxford, as we have before pointed out, stands at the head of an orderly and dignified ecclesiasticism. It is well for a progressive civilisation, more than well indeed, that it should have a religion great in its leading principles, and beautiful as to its outward forms. But when such a religion has nothing to recommend it but its forms and its logic, the gross world is apt to despise it, and generous enthusiasts are tempted to sweep it away. The sight of such an antiquated infirmary as the Radcliffe at Oxford, and the knowledge that sanitary science elsewhere was doing beneficent work with which the deficient resources of the Radcliffe Infirmary could by no means compete, roused our Special Commissioner some time ago to much energy of feeling and to the use of language which could not be described as other than strong. Now, however, the time for strong language is past; the Oxford managers have resolved upon their scheme of renovation: they have made their appeal to their constituents and neighbours, and they hopefully await the result. The very best intentioned managers cannot make bricks without straw: the bravest and most successful of generals are powerless without men and money. For our part we feel that it is now as much our duty to stand side by side with the managers as it was formerly our duty to assail them from every available fighting point. If the Radcliffe Infirmary should remain long without enlargement and needed sanitary improvement, it will not be the fault of the managers or of the medical staff. Oxford is rich, and the county is rich: the very modest sum of £10,000 is asked for. Enthusiastic and far-seeing lovers of hospitals would have been better pleased if Dr. Bright and his Committee had asked for ten times ten thousand; if they had razed their old infirmary to the ground, and built a noble structure worthy of the other beautiful and striking buildings which constitute the outward and visible embodiment of the world's most famous University. But if the hundred thousand is not to be forthcoming ten thousand ought to be subscribed by the county, the city, and the University, with the utmost cheerfulness and promptitude. It is but natural that the rich should give of their comparative abundance. But it is neither wise nor right that they should be asked to do all. They will set the example of liberal giving, no doubt; and it will be for the middle classes, the farmers, the tradesmen, and even the artisans and the labourers, to follow suit and take their share in the great, benevolent, and truly religious work. We commend the movement to all our readers with the utmost cordiality; and we hope Dr. Bright and the other managers of the infirmary will accept our sincere congratulations on their resolution to render to Oxford and the county one of the greatest public services which modern civilisation can demand.

